# THE ROLE OF RELIGIOSITY IN DEPRESSIVE SYMPTOMS AMONG CHINESE OLDER ADULTS

**DOI:** 10.1093/geroni/igz038.3537

**Published:** 2019-11-08

**Authors:** Ru Jia, Dexia Kong, XinQi Dong

**Affiliations:** Rutgers University, New Brunswick, New Jersey, United States

## Abstract

This study aims to examine the relationship between religiosity and depressive symptoms in a large cohort of community-dwelling U.S. Chinese older adults living in the Greater Chicago area, which has received relatively little research attention. Cross-sectional self-report data was obtained from the Population Study of Chinese Elderly in Chicago between 2011 and 2013 (N=3,157). Depressive symptoms were measured by the nine-item Patient Health Questionnaire (PHQ-9). A score of 5 and above indicated the presence of clinically significant depressive symptoms. Logistic regression analyses were conducted to examine the association between religiosity and depressive symptoms. Out of 3,157 participants, 20.3% participants had a score of or above 5 on PHQ-9. 35.4% reported religiosity as being “important” (24.7%) and “very important” (10.7%); 16% reported attending organized religious services at least once a month (3.1% reported once a month; 12.3% reported once a week; 0.6% reported almost every day); 23% reported having religious services at home at least once a month (10.3% reported once a month; 3.2% reported once a week; 9.5% reported almost daily). Results showed that recognizing religiosity as important is significantly negatively associated with depressive symptoms (odds ratio [OR]=0.94, 95% confidence interval [CI]=0.89-0.99). However, no significant associations between depressive symptoms and religious activity attendance or religious service at home were observed. Findings suggest that senses of belonging and life meaning may help reduce depressive symptoms, rather than the religious activities per se. Future interventions could reduce depressive symptoms of U.S. Chinese older adults through religiosity.

